# Synthesis of Metakaolin Based Alkali Activated Materials as an Adsorbent at Different Na_2_SiO_3_/NaOH Ratios and Exposing Temperatures for Cu^2+^ Removal

**DOI:** 10.3390/ma16031221

**Published:** 2023-01-31

**Authors:** Masdiyana Ibrahim, Wan Mastura Wan Ibrahim, Mohd Mustafa Al Bakri Abdullah, Marcin Nabialek, Ramadhansyah Putra Jaya, Monthian Setkit, Romisuhani Ahmad, Bartłomiej Jeż

**Affiliations:** 1 Faculty of Chemical Engineering & Technology, Universiti Malaysia Perlis (UniMAP), Arau 02600, Malaysia; 2 Center of Excellence Geopolymer & Green Technology (CeGeoGTech), Universiti Malaysia Perlis (UniMAP), Kangar 01000, Malaysia; 3 Faculty of Mechanical Engineering & Technology, Universiti Malaysia Perlis (UniMAP), Arau 02600, Malaysia; 4 Department of Physics, Częstochowa University of Technology, 42214 Częstochowa, Poland; 5 Faculty of Civil Engineering Technology, Universiti Malaysia Pahang, Kuantan 26300, Malaysia; 6 School of Engineering and Technology, Walailak University, Nakhon Si Thammarat 80160, Thailand; 7 Department of Technology and Automation, Faculty of Mechanical Engineering and Computer Science, Czestochowa University of Technology, 42200 Czestochowa, Poland

**Keywords:** adsorption, alkali activated materials, waste water, exposing temperatures, removal

## Abstract

Water contamination is a major issue due to industrial releases of hazardous heavy metals. Copper ions are among the most dangerous heavy metals owing to their carcinogenicity and harmful effects on the environment and human health. Adsorption of copper ions using alkali activated materials synthesized through the polycondensation reaction of an alkali source and aluminosilicates is the most promising technique, and has a high adsorption capability owing to a large surface area and pore volume. This research focuses on the effect of the alkaline activator ratio, which is a sodium silicate to sodium hydroxide ratio. Various exposing temperatures on metakaolin based alkali activated materials on a surface structure with excellent functional properties can be used as adsorbent materials for the removal of copper ions. A variety of mix designs were created with varying sodium silicate to sodium hydroxide ratios, with a fixed sodium hydroxide molarity, metakaolin to alkali activator ratio, hydrogen peroxide, and surfactant content of 10 M, 0.8, 1.00 wt%, and 3.0 wt%, respectively. Most wastewater adsorbents need high sintering temperatures, requiring an energy-intensive and time-consuming manufacturing process. In this way, metakaolin-based alkali activated materials are adsorbent and may be produced easily by solidifying the sample at 60 °C without using much energy. The specific surface area, water absorption, microstructure, phase analysis, functional group analysis, and adsorption capability of copper ions by metakaolin based alkali activated materials as adsorbents were evaluated. The water absorption test on the samples revealed that the sodium silicate to sodium hydroxide 0.5 ratio had the highest water absorption percentage of 36.24%, superior pore size distribution, and homogeneous porosity at 60 °C, with a surface area of 24.6076 m^2^/g and the highest copper ion uptake of 63.726 mg/g with 95.59% copper ion removal efficiency at adsorption condition of pH = 5, a dosage of 0.15 g, 100 mg/L of the initial copper solution, the temperature of 25 °C, and contact time of 60 min. It is concluded that self-supported metakaolin based alkali activated material adsorbents synthesized at low temperatures effectively remove copper ions in aqueous solutions, making them an excellent alternative for wastewater treatment applications.

## 1. Introduction

The existence in industries of toxic metals created by mineral processing causes a significant threat to the water environment [1]. As a result, serious rules are necessary for many countries to effectively recover harmful metal ions from wastewater prior to their fast and uncontrolled discharge into the natural environment. Metal ions are often non-biodegradable compounds, and large concentrations may harm humans, animals, and the environment. The accumulation of heavy metals in the human body as a consequence of long-term exposure, for example, may cause major health concerns such as organ malfunction, Alzheimer’s disease, and anaemia [2,3,4]. Therefore, to overcome the water cleaning environment, removing metal ions from industrial wastewater is important to minimize amounts of heavy metals.

Among the heavy metals, copper is widely used in industry. It is accumulated in waste streams from such industries as mining, electrical equipment, batteries and wires, pipes, paper and fertilizers, alloys, antifouling paints, and wood preservatives [5,6]. Copper is a persistent, bio-accumulative, and toxic chemical, so it is considered the second most toxic metal to aquatic organisms after mercury [7,8,9]. Long-term and continuous exposure to copper and nickel in the body causes nausea, vomiting, headaches, diarrhea, respiratory problems, liver and kidney failure, nervous system damage, cancer, and death in humans [10,11,12,13]. The US Environmental Protection Agency (USEPA) and the World Health Organization (WHO) established the allowable Cu (II) ion in water levels at 1.3 mg/L and 2.0 mg/L, respectively, due to their major effects [14,15,16]. Since they are nondegradable and have the propensity to bioaccumulate, it is important to find efficient ways to eliminate them from the environment.

Chemical precipitation, reverse osmosis, coagulation, ion exchange, electrochemical treatment technologies, and membrane filtration are recent methods for removing metal particles from wastewater [17,18,19]. Chemical and electrical technologies, on the other hand, are low efficiency, have a high energy requirement, require toxic substance precipitation, and are cost-ineffective [20,21]. As a result, using locally available materials as adsorbents for copper ion removal is becoming extremely prevalent. Adsorption is a flexible method of separating a solute from a solution by attaching it to the surface of an adsorbent that delivers high-quality treated effluent in various situations [20,22,23,24,25].

Various materials, such as zeolites, active carbon, biomaterials, and clay minerals may be utilized as adsorbents for organics and metal ions to filter wastewater [26,27]. Alkali activated materials (AAMs), or geopolymers that are prepared from precursor aluminosilicates, are a newer technology for heavy metal adsorption from wastewater, composed of silica and alumina near zeolite material to replace conventional adsorbents [12,28]. Alkali activated materials (AAMs) are more environmentally friendly than other materials due to their sustainable production process, which involves less energy usage and less waste stream or byproduct consumption [29]. Between the tetrahedron [AlO_4_] and [SiO_4_] units, AAMs are a kind of cross-linked long-chain inorganic polymer material with outstandingly high strength, corrosion resistance, fire resistance, and long life properties [30,31]. AAMs are porous amorphous gels that may also serve as adsorbents, allowing them to absorb heavy metal ions [32,33]. In addition, porous adsorbents are the most efficient, dependable, straightforward, and cost-effective sorption [34,35,36]. AAM adsorbents have a significant sorption capacity in determining their effectiveness in wastewater treatment for a variety of heavy metals, due to their unique physicochemical features, such as surface chemistry, porous structure with both high internal surface area and porosity, and long-lasting, eco-friendly cementitious material [26,37,38].

Subsequently, the chemical and mineralogical composition of the raw materials, the kind of alkaline activators, their concentration, and the ratio of main chemical activators such as Na_2_O/SiO_2_, SiO_2_/Al_2_O_3_ are all parameters that influence alkali activation [39,40,41]. In that sense, the main objective of this study is to study the porous distribution behaviour for heavy metal adsorption. The optimization of mixing parameters, which is the sodium silicate to sodium hydroxide (Na_2_SiO_3_/NaOH) ratio of metakaolin based alkali activated materials, was investigated as mixing formulation and is known to crucially affect the physical and mechanical properties of geopolymer. The optimum mixing parameter was selected based on the highest percentage of water absorption; the relationship between water absorption and porosity increases exponentially as the pore size of the structure grows [42,43].

In general, the excellent copper ion adsorption capacities in AAMs are influenced by physical characteristics such as surface area, pore volume, and pore size distribution. These unique characteristics depend on the type of raw materials employed for the preparation of AAM adsorbents and the method of activation. Much recent research has focused on generating adsorbent materials with an interconnected cellular network using a saponification or gel-casting combination approach. However, the abovementioned techniques are time-consuming and unsuitable for mass production [44,45]. A directly pore-forming agent was mixed with a surfactant and a specific catalytic acid component to make hybrid foams with interconnected porosity and low density. As a chemical blowing agent for producing high porosity components, hydrogen peroxide (H_2_O_2_), which produces air or gas via direct foaming, is increasingly gaining popularity. This novel material is employed in wastewater treatment systems due to the high copper removal effectiveness of the adsorption approach, which eliminates the need for large volumes of commercial inorganic precipitants, produces no sludge, and does not require the use of support materials. Furthermore, the prepared metakaolin based alkali activated materials were discovered to be highly effective separable materials for the selective removal of Cu(II) ions from water samples, and were considered to be the most efficient, dependable, simple, and economical adsorbents for the sorption that presented improved capacities of Cu(II) ions. Therefore, this study synthesised novel metakaolin based alkali activated materials and conjugated onto porous metakaolin based AAM adsorbents for simultaneous hazardous Cu(II) ion detection and removal from contaminated waste samples. The ligand of metakaolin based AAM adsorbents was acknowledged as having an extremely large surface area and huge pore volumes, implying a promising adsorbent material for Cu(II) ion capture from an aqueous solution. Metakaolin based alkali activated materials can detect the Cu(II) ion and adsorb with a greater adsorption capability than other materials.

## 2. Methodology

### 2.1. Materials

#### 2.1.1. Metakaolin

The metakaolin with the total SiO_2_ and Al_2_O_3_ composition of 91.4 wt% was obtained at 850 °C at a 5 °C/min heating rate with the calcination of 2 h of kaolin. The used kaolin was purchased from Kaolin (Malaysia) Sdn. Bhd., Bidor, Perak, Malaysia. The internal composition of the kaolin used as powder had an average of 2% moisture density, at least 40% lower than 2 μm.

#### 2.1.2. Sodium Hydroxide

The sodium hydroxide (NaOH) solution was made by dissolving a 99% pure NaOH pellet supplied by Brenntag Sdn. Bhd., Shah Alam, Selangor, Malaysia, in 1000 mL of distilled water in a volumetric flask at a concentration of 10 M. As researched by Ain et al. [46], 10 M was selected as the concentration of NaOH to be fixed, which has high porosity and low density with suitable compressive strength to be utilized for metakaolin based alkali activated materials as the copper ions adsorbent in this research.

#### 2.1.3. Sodium Silicate

The Na_2_SiO_3_ (technical grade) was provided by South Pacific Chemical Industries Sdn. Bhd. (SCPI) of Malaysia, with a composition of 30.1% SiO_2_, 9.4% Na_2_O, and 60.5% H_2_O (SiO_2_/Na_2_O ratio of 3.20). Na_2_SiO_3_ is a colourless fluid that dissolves readily in water.

#### 2.1.4. Foaming Agent

To produce metakaolin based alkali activated materials as adsorbent for copper removal, 1.00 wt% by mass of metakaolin of hydrogen peroxide (H_2_O_2_) was added as a foaming agent that was intended to develop pores inside the specimen. A 3 wt% hydrogen peroxide (diluted from 30 wt% H_2_O_2_, Sigma Aldrich, Malaysia) solution was formulated for high stability and long-term storage with strong oxidizing properties. Moreover, Tween 80 or polysorbate 80 at a composition of 70% oleic acid (balance primarily linoleic, palmitic, and stearic acids) from Sigma Aldrich, Malaysia was mixed as a surfactant and is commonly used as a function to decrease surface tension and drainage of alkali activated materials.

### 2.2. Methods

#### 2.2.1. Preparation of Metakaolin Geopolymer Adsorbents

Metakaolin powder was mixed with an alkaline activator solution containing sodium hydroxide (NaOH) and sodium silicate (Na_2_SiO_3_) at a fixed 0.8 solid-to-liquid ratio, by mass, which was selected based on previous studies. The sodium silicate to sodium hydroxide ratio in this investigation was set at 0.25, 0.5, 0.75, 1.0, and 1.25. The molarity used for sodium hydroxide was fixed at 10 M, which had been left at room temperature for 24 h. For the first stage, hydrogen peroxide content was fixed at 1.00 wt% by mass of solid and 3.0 wt% of Tween 80 by mass of solid as a surfactant and was successively added to the suspension. The mixture was mixed using a mechanical stirrer until it became a homogenous paste. The metakaolin based alkali activated materials were formed into the required shape and size, a sphere shape of 1–2 cm. The samples were put in a tray to keep inside the oven. In addition, the fresh geopolymer paste from each parameter prepared was poured into high-density polyethylene (HDPE) mould with the dimensions of 50 mm × 50 mm × 50 mm for compressive strength testing. The moulded samples were vibrated for 2 min on the vibration table to remove entrapped air. The tray was covered with wrapping plastic to prevent the loss of humidity in the metakaolin-based AAMs. Then, the samples were cured at 60 °C for 24 h for polycondensation during the alkali activation process. Next, the selected mix design was prepared with various exposing temperatures. The samples were sealed with wrapping plastic to prevent moisture loss on metakaolin based alkali activated materials. The samples were evaluated at several curing temperatures, including room temperature (25, 60, 100, 500, and 900 °C) to obtain excellent characteristics of metakaolin-based alkali activated materials as adsorbent for copper ions. The samples were kept sealed at room temperature for 7 days before testing. This process underwent crush and sieve through a 150 μm particle size sieve to become powder adsorbent. The smaller the adsorbent particle size, the larger the surface area. Smaller particles also reviewed can increase the effectiveness of adsorption.

#### 2.2.2. Water Absorption Test

Water absorption was conducted according to the standard research methodology of ASTM C 642 [47]. Three samples were absorbed in water at room temperature for 24 h. Then, the samples were removed with a wet cloth and allowed to drain before the saturated weight was recorded. After that, the sample was dried first in the oven at a temperature of 105 °C for 24 h and then the oven dry weight of the metakaolin based AAM was recorded. Water absorption was determined by Equation (1) below:

(1) $$\mathrm{Water}\;\mathrm{absorption}\;(\%)=\frac{\mathrm{Ws}-\mathrm{Wd}\;}{\mathrm{Wd}}\times 100\%$$

where W_s_ represents the saturated weight, which is the weight of metakaolin based AAMs after 24 h in the water, and W_d_ represents the original dry weight (g).

#### 2.2.3. Density Test

The density test was examined by densimeter to measure the weight of metakaolin based AAMs in air and water following ASTM D792 [48]. The specific gravity, or density in gram per cubic centimetre unit of a solid, is a property that can be conveniently measured to identify a material, track physical changes in a sample, indicate the degree of uniformity among different sampling units or specimens, or determine the average density of an item. The samples were kept at room temperature for a total of 7 days until the curing procedure was completed before the test could be completed successfully. Three samples of each metakaolin based AAMs were measured, and an average of three results were reported.

#### 2.2.4. Compressive Strength Test

Compressive strength measures the maximum compressive load a material can bear before fracturing. It is an important performance that provides an indicator of its quality and determines the strength developed by the alkali activated materials. Thus, the performances of alkali activated materials with various sodium silicate to sodium hydroxide ratios (Na_2_SiO_3_/NaOH), curing, and high heat treatments on metakaolin based alkali activated materials was determined in terms of compressive strength. The compressive strength were evaluated using a Universal Testing Machine (UTM), Shimadzu Japan, UH-1000 kN, with a loading rate of 0.5 MPa/s. Compressive strength was performed on cubes with 50 × 50 × 50 mm^3^ according to ASTM C109 [49]. The samples were tested at 7 days, and already had their early strength. Three specimens were tested to determine the compressive strength of metakaolin based AAMs, and the mean value of the three samples was reported.

#### 2.2.5. Characterization of Metakaolin Based Alkali Activated Materials

The chemical composition of raw material was determined by using PANanalytic PW4030 X-ray fluorescence (XRF) spectrometer with model type MiniPAL-4 (Malvern Panalytical, Worcestershire, UK) using an energy dispersive microprocessor controlled analytical instrument. The sample was loaded in the spectrometer chamber and operated at a maximum current of 1 mA to generate X-ray and a maximum voltage of 30 kV to stimulate the sample. Scanning electron microscopy (SEM) was conducted for morphology analysis using TESCAN VEGA’s 4th generation Scanning Electron Microscope (SEM) (Brno, Czech Republic) with a tungsten filament electron source and combines SEM imaging. All sample preparation is solid and coated with gold using Sputter Coater NS800 model. The signals generated during analysis produced a two-dimensional image and revealed information about the sample, including the orientation of materials making up the sample and the external morphology (texture) at a magnification level of ×500 scanning voltage. Phase analysis to investigate the crystalline material structure, including atomic arrangement, crystalline size, and imperfections, was tested by using XRD powder diffractograms. Metakaolin based AAMs at different curing and heat exposure at elevated temperature samples were analyzed using a XRD-6000 Shimadzu X-Ray Diffractometer (Columbia City, IN, USA) with Cu Kα radiation produced at 30 mA and 40 kV. The XRD data were collected at 2θ values from 10 to 80°. The specific surface area, pore volume, and pore size distribution were determined by the Brunauer, Emmett and Teller (BET) and Barrett, Joyner, and Halenda (BJH) methods, respectively, using a Micrometrics Tristar II 3020 (Micromeritics Instrument Corporation, Norcross, GA, USA) volumetric adsorption/desorption apparatus. Following ASTM D3663-20 [50], the amount of nitrogen gas adsorbed by the sample at different low-pressure conditions was used to measure the surface area of metakaolin AAMs. The degassing of nitrogen gas (N_2_) adsorption–desorption isotherm was passed through the adsorbents at a liquid nitrogen temperature of 200 °C for 4 h. For analyzing the elemental distribution, microbeam energy dispersive X-ray fluorescence spectroscopy (μ-XRF) was performed at the Beamline 6 of the Synchrotron Light Research Institute (SLRI), Bangkok, Thailand. At Beamline 6, continuous synchrotron radiation with an energy exceeding 2 keV and a beam diameter of 100 μm was employed. The samples were positioned vertically on the holder, and high-precision motorized stages were used to carry out the raster scanning. The experiment was carried out with a total scan of 441 values in a helium gas atmosphere. PyMca software was used to demonstrate the micro-XRF data.

#### 2.2.6. Adsorption of Copper Ions Test

The metakaolin based alkali activated materials (AAMs) adsorbent was washed with distilled water for about 1 h to avoid the effect of precipitation and excesses amount of sodium hydroxide on the samples, and dried under vacuum at 60 °C for 24 h. Then, 0.15 g of dried samples of metakaolin based AAMs adsorbent at different curing and heat treatment conditions were tested with 100 mg/L copper nitrate solution fixed at pH 5. This adsorption test parameter followed Cheng et al., [51] and Ge et al. [52], and resulted in high copper adsorption capacity and high removal efficiency by metakaolin geopolymer adsorbent. The Erlenmeyer flasks were placed into an orbital shaker at room temperature, ~25 °C, with a shaking speed of 250 shakes/min for 1 h. Samples were taken and analyzed for Cu^2+^ concentration using atomic absorption spectroscopy, AAS (Perkin Elmer, Llantrisant, UK). The experiment was repeated three times for each condition to obtain the mean values. Five standard solutions comprising 0, 0.5, 1.0, 1.5, and 2.0 mg/L of copper ion solution were used to calibrate the AAS instrument, and the calibration curve’s correlation coefficient was greater than 0.9999. Further, sample solutions with complex matrices were not used, and no apparent matrix interference was observed.

## 3. Results and Discussion

### 3.1. Raw Material Characterization

In this investigation, X-ray fluorescence (XRF) examination was used to portray the synthetic arrangement of every precursor raw material. Every composition of metakaolin used is summed up by the XRF investigation result shown in Table 1.

Table 1 shows that the main constituents of metakaolin are Si and Al in structure, with the chemical composition containing silica (SiO_2_) and alumina (Al_2_O_3_), which are 56.7 wt% and 34.7 wt%, correspondingly. There were small impurities of titanium (TiO_2_), iron (Fe_2_O_3_), and phosphorus (P_2_O_5_) that equal to 3.13 wt%, 2.09 wt% and 1.68 wt%, respectively. The total of these compositions is about 98.3 wt% and this indicates that this metakaolin powder is pozzolans which exceed 70 wt%, as suggested for pozzolans to have a good pozzolanic activity according to the ASTM C 618 standard specifications [53]. In brief, pozzolanic materials, finely split siliceous particles or a combination of siliceous and aluminous materials, react with calcium hydroxide (CH) during cement hydration to generate calcium silicate hydrate (C-S-H) with excellent microstructure and mechanical cementitious properties [54,55,56,57]. There was likewise a little hint of calcium (CaO), potassium (K_2_O), zirconium (ZrO_2_), strontium (SrO), gallium (Ga_2_O_3_), and copper (CuO); it was not so much critical of the fact that it had under 1 wt%.

Since the alkali activated materials are skeletal structures that form from the polycondensation of aluminosilicate materials, high silica, and alumina materials are appropriate for binding action materials [58,59]. This rigid structure is the outcome of a process that involves the complete dissolution of the aluminosilicate phase in alkali solution, which generates two separate tetrahedral ends made of aluminosilicate, silicates (SiO_4_), and aluminates (AlO_4_) that are joined by oxygen atoms. Accordingly, two major phases, which are aluminosilicate dissolution and separation into alumina and silicate end, and the polycondensation/polymerization stage, are likely to be produced based on the ratio of SiO_2_ to Al_2_O_3_ in the raw material [20,40]. Therefore, the proportion of [SiO_4_]^4−^ to [AlO_4_]^5−^ is one of the relevant metrics for evaluating the alkali activation process and the structural properties of the resulting alkali activated materials. With higher Al and Si but lower Ca, the supersaturating period is achieved at a longer time, allowing better workability and greater reactivity [60,61]. Furthermore, Zahid et al. [60] reported the segments of Si and Al to add to the creation of geopolymer power by geopolymerization of Al-Si, which happens because of the utilization of alkaline activators and effective curing.

**Figure 1:**
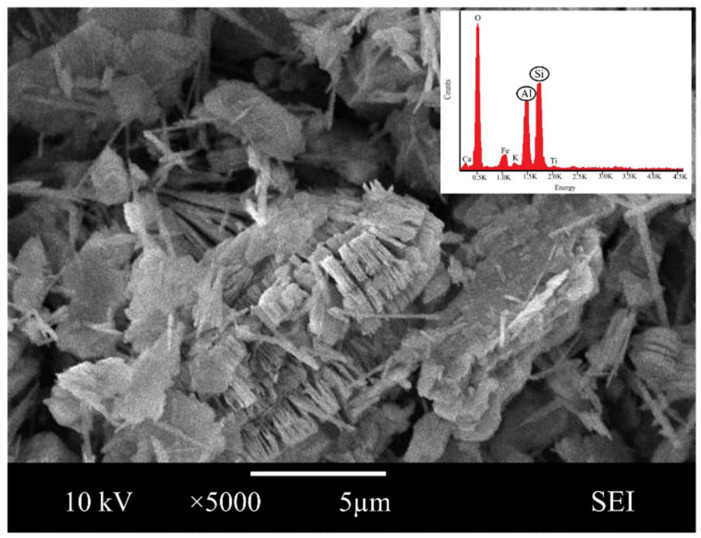
SEM images and energy dispersive X-ray analysis (EDX) of metakaolin.

Scanning electron microscope analysis revealed the morphological form of pure crystal in magnification at ×5000. The SEM image showed the characteristic morphology of the original metakaolin in Figure 1, indicating that metakaolin is a heterogeneous material possessing a dense matrix of irregularly shaped and flake-like particles.

Similar observations were found by Jaya et al. [62,63], who presented that the metakaolin has a flake-like structure, and Alouani et al. [64], who presented that the obtained metakaolin is a heterogeneous material and consists of irregularly shaped particles. Flaky particles of metakaolin observed provided a larger surface area and availability of more contact area with water. This is also in line with Kara et al. [26], who reported that metakaolin has a large specific surface area, and its particles are in flake shape.

Moreover, the elemental composition by Energy dispersive X-ray, EDX showed that the major structure of metakaolin is made up of oxygen (51.63%), silica (23.06%) and alumina (21.64%). Given the compound synthesis of metakaolin, the result reveals that they have a significant measure of the content of silica and alumina, a substance rich in silica and alumina, that can be categorized as a geopolymer source material.

### 3.2. Effect on Na_2_SiO_3_/NaOH Ratio to Metakaolin Based Alkali Activated Materials

#### 3.2.1. Water Absorption Analysis

**Figure 2:**
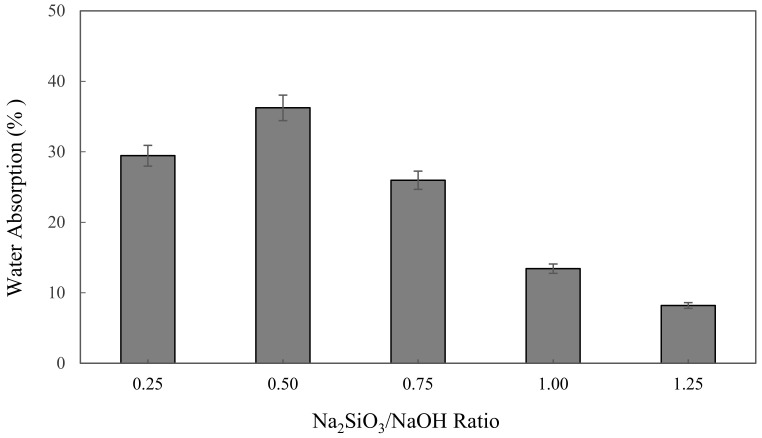
The water absorption of metakaolin based alkali activated materials with different Na_2_SiO_3_/NaOH ratios.

The water absorption of alkali activated metakaolin in units of percent (%) is depicted in Figure 2. The highest water absorption was found at 0.5 Na_2_SiO_3_/NaOH ratio of 36.24%, while the lowest was at 1.25 of Na_2_SiO_3_/NaOH ratio of 8.2%. The figure shows that the water absorption slightly increased from 29.45% to 36.24%, as observed with increases in Na_2_SiO_3_/NaOH ratio from 0.25 to 0.5. However, the water absorption was decreased with the increase of the Na_2_SiO_3_/NaOH ratio from 0.5 to 1.25, which dropped by 28.04% to its lowest point at 8.2%.

A decline in water absorption when increasing the sodium silicate to sodium hydroxide ratio influenced the workability of metakaolin based alkali activated materials. Workability was identified as a property of fresh binder, which measures the ease with which the fresh paste can be mixed, placed, consolidated, and finished. The increase in the Na_2_SiO_3_/NaOH ratio was induced to decrease the workability and porosity of metakaolin AAMs as liquid sodium silicate, generating high viscosity and density compared to NaOH solution, concurrently improving the sodium silicate content, which reduced the workability and porosity of alkali activated metakaolin.

Porosity gives materials important properties, such as low density (meaning lightweight) and a large surface area to store molecules in the pores in line with these properties, which are suitable for making AAM as adsorbent materials. Hossain et al. [65] mentioned that water absorption mainly depends on capillary pore volume, and the volume of artificial pores governs the compressive strength and density of the alkali activated materials.

#### 3.2.2. Density and Compressive Strength Analysis

**Figure 3:**
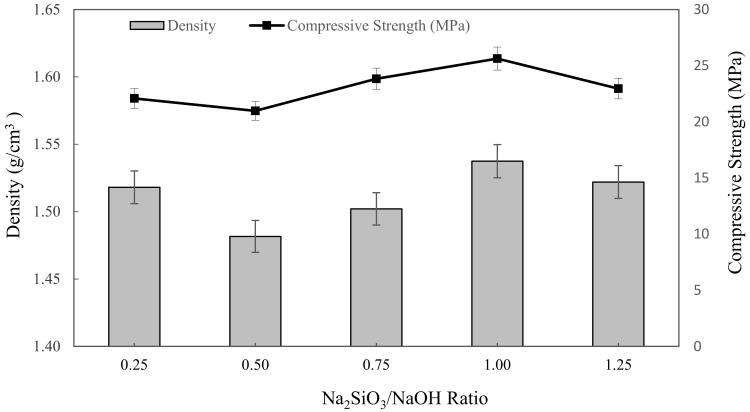
The density and compressive strength of metakaolin based alkali activated materials with various Na_2_SiO_3_/NaOH ratios.

The densities of metakaolin based alkali activated materials with different Na_2_SiO_3_/NaOH ratios are shown in Figure 3. Overall, the bulk density slightly decreased with increasing mixing parameters, up from 0.25 to 0.5 Na_2_SiO_3_/NaOH ratio, then climbed until it reached the highest density with 1.5375 g/cm^3^ at 1.00 of Na_2_SiO_3_/NaOH ratio. The lowest density of alkali activated metakaolin was depicted at 0.5 of the Na_2_SiO_3_/NaOH ratio, which is 1.4816 g/cm^3^. These density values were comparable to those reported by Kwek et al. [66] for POFA geopolymer, which ranged from 1.658 to 1.741 g/cm^3^, and Hwang et al. [67] for fly ash and residual rice husk ash-based geopolymers, which were 2.18 and 2.08 g/cm^3^, respectively.

According to this finding, the alkaline activator ratio of 0.50 had the least influence on density. This might be a result of the highest water absorption with high porosity in the samples. Boke et al. [68] also found that the density of the synthesized foamed alkali activated materials increased with decreasing water absorption. Dehydroxylation and crystallization phases formed during the alkali activation, bonding, and the presence of a hydroxylase group (–OH) also influenced the samples’ densities. Hence, the increasing density of alkali activated metakaolin exceeds 0.5 up to 1.0 Na_2_SiO_3_/NaOH ratio due to the high dissolution of the metakaolin particles in the mixture obtained by higher alkali content, resulting in denser microstructure in geopolymers. Furthermore, increasing sodium silicate content aided in the creation of alkali activated materials networks as well as the condensation process of alkali activated metakaolin, resulting in higher density. This conclusion also was supported by Alharbi et al. [69] and Dogan-Saglamtimur et al. [69].

Gradually, the compressive strength of metakaolin based alkali activated materials depended on the water absorption and density of alkali activated materials. In general, the graph shows that with increasing mixing parameters, the compressive strength declined slightly at 0.25 to 0.5 Na_2_SiO_3_/NaOH ratio, then increased as the Na_2_SiO_3_/NaOH ratio increased up to 1.25. The highest compressive strength was obtained by a 1.25 Na_2_SiO_3_/NaOH ratio of 26.3625 MPa, while the minimum strength was obtained by a 0.5 Na_2_SiO_3_/NaOH ratio of 20.975 MPa. This range of compressive strength value is acceptable for metakaolin based alkali activated materials as an adsorbent. However, for the alkali activated metakaolin with Na_2_SiO_3_/NaOH ratio above 0.5, the alkali activation or geopolymerization was not favoured, and led to the formation of a sticky mixture and increased the viscosity of alkali activated materials. This was attributed to the obvious inclusion of Na_2_SiO_3_, which served as a coagulant to speed up dissolution during the alkali activation process and also served as a polymerization intermediate or plasticizer and was denser, and resulted in an increase in compressive strength when the alkaline activator ratio was increased.

#### 3.2.3. Microstructure Analysis

**Figure 4:**
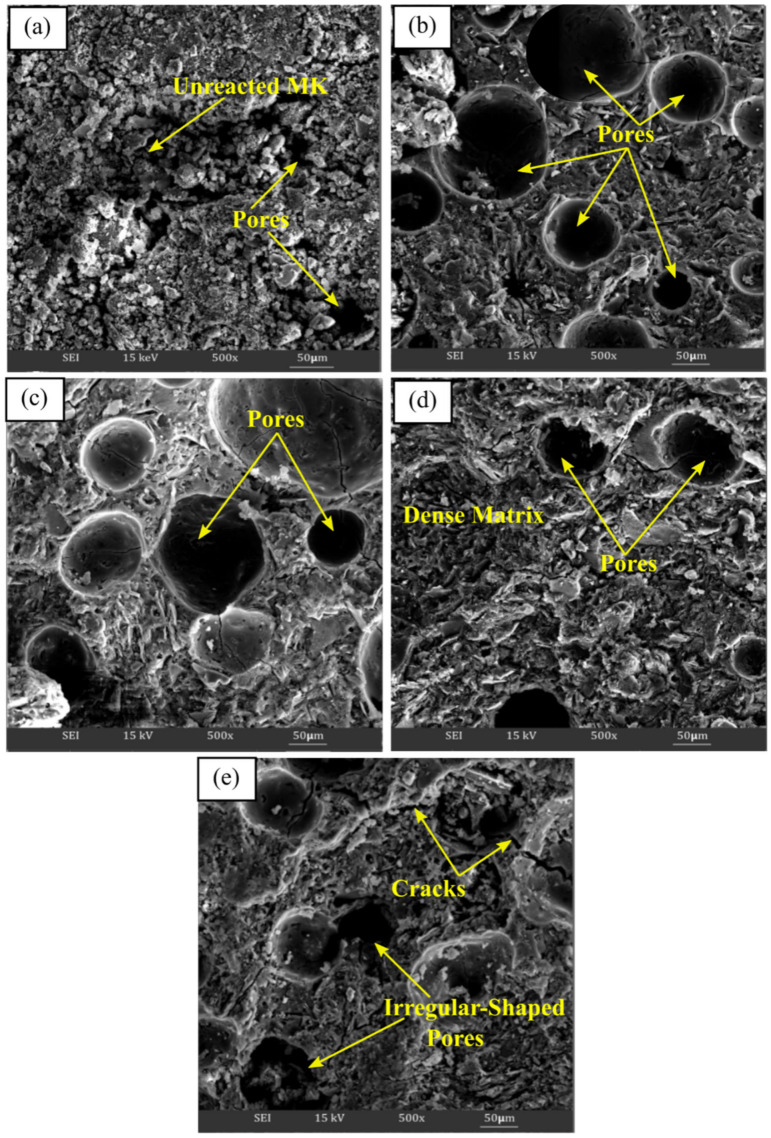
SEM micrograph of metakaolin based alkali activated materials at (**a**) 0.25, (**b**) 0.50, (**c**) 0.75, (**d**) 1.00, and (**e**) 1.25 of Na_2_SiO_3_/NaOH ratios.

Figure 4a–e illustrate the SEM micrograph of metakaolin based alkali activated materials activated with various Na_2_SiO_3_/NaOH ratios at ×500. The microstructure of metakaolin based alkali activated materials at the lowest Na_2_SiO_3_/NaOH ratios of 0.25 exhibited markedly different microstructures compared with other ratios. It had loose matrices with a large amounts of non-reacted and partially reacted metakaolin particles embedded in poor matrices, as shown in Figure 4a. Then, the existence of pores contributed to the porous structure, where they were largely affected and became lesser, or changed to irregularly shaped pores, as the Na_2_SiO_3_/NaOH ratio increased, which increased the density of the metakaolin based alkali activated materials.

Figure 4b with 0.5 Na_2_SiO_3_/NaOH ratio revealed a more homogenous and continuous microstructure, with regular pore shape distribution, which, as discussed, demonstrated the highest percent of water absorption. In general, all the alkali activated metakaolin have a generally homogeneous microstructure at increasing Na_2_SiO_3_/NaOH ratios of 0.50 up to 1.00. The presence of unreacted metakaolin particles was reduced, and the matrices seemed to be dense, indicating greater binding between metakaolin and the alkaline activator solution, resulting in an increase in compressive strength.

However, owing to the inadequate interaction of soluble species with metakaolin particles with increasing water glass content, a high Na_2_SiO_3_/NaOH ratio of 1.25 causes the matrices to become less dense with less aluminosilicate matrix. This will result in poor metakaolin particle disintegration and huge, irregularly shaped pores. Cracks also appeared due to the incomplete reaction that occurred between the precursor materials and the activator at the selected zone. As a result, the density and compressive density of the structure declined.

### 3.3. Effect on Different Exposing Temperature to Metakaolin Based Alkali Activated Materials

#### 3.3.1. Water Absorption Analysis

**Figure 5:**
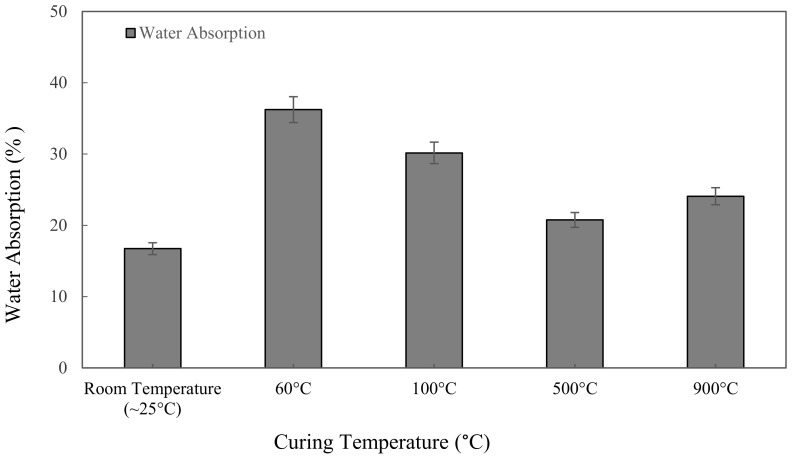
Water absorption of metakaolin based alkali activated materials at different exposing temperatures.

Figure 5 shows the water absorption values for the metakaolin based alkali activated materials with different curing and high-temperature exposure, tested after 7 days. Samples cured at 60 °C for 24 h give the highest value of water absorption at 36.24%. Due to their greater apparent porosity, specimens that were cured at 60 °C showed the highest water absorption value. The lowest water absorption, 16.73%, was discovered when samples were kept at room temperature for 24 h. Figure 5 shows the water absorption of metakaolin based alkali activated materials increases with increasing the curing temperature from ~25 °C to 60 °C, and declined when the samples were exposed at 100 °C and 500 °C, but then went up to 24.08% when exposed to 900 °C.

A decrease in water absorption from 36.24% to 20.76% was observed with increased heat temperature exposure from curing at 60 ℃ to exposure at high temperature 500 °C. Regardless of the high heat exposure approach from 500 to 900 °C, the water absorption of alkali activated metakaolin dropped as the temperature rose. The reduction is due to high heat shrinkage, which causes the hardened structure to become more compact, lowering its permeable porosity. Meanwhile, when the temperature is exposed to 900 °C, the microcracks and cracks in the samples become wider due to the high-temperature exposure, increasing water absorption into the binder matrix. Samal et al. [70] claimed that thermal cracking was attributable to a faster rate of geopolymerization in the samples, resulting in extreme expansion when exposed to high temperature.

#### 3.3.2. Density and Compressive Strength Analysis

**Figure 6:**
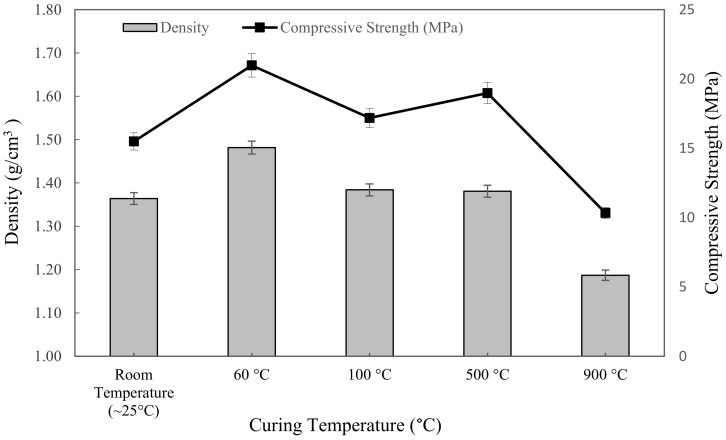
The density and compressive strength of metakaolin based alkali activated materials at different exposing temperatures.

Figure 6 showed the density of metakaolin based alkali activated with various exposing temperatures and tested after 7 days. The trend of the density line graph was opposite to the water absorption. The highest density was found in the samples cured at 60 °C, which was 1.4816 g/cm^3^, while the lowest was the sample sintered at 900 °C, with 1.187 g/cm^3^. At first, the increment in density was exhibited as increasing curing temperature from ~25 °C to 60 °C. Then, as can be seen, it slightly went down with an increasing curing temperature of 100 °C, and that led to a drop when exposed to 900 °C.

In general, raising the temperature from room temperature to 60 °C considerably increased the density because the samples would give more dissolution of the metakaolin, which would densify the microstructure of the geopolymer, as opposed to exposing it to a ~25 °C initial curing temperature. The density of metakaolin based alkali activated materials cured at 60 °C was more uniform than that of specimens exposed to higher temperatures of 100 °C, 500 °C, and 900 °C. This might be due to the N-A-S-H gel forming more effectively, resulting in a homogenous pore structure and absence of cracks at 60 °C curing temperature.

Likewise, due to the presence of excess water from the foaming agent, the high temperature caused the water to evaporate, which, consequently, produced cracks and reduced the density of metakaolin based alkali activated materials. This is because the foaming agent, which is hydrogen peroxide and tween 80 as a surfactant, was not involved in alkali activation process. Skoczylas et al. [71] also reported that increasing the exposure temperature caused the disintegration in the bonding network as well as the expansion of the specimens, which was attributed to the reduced geopolymerisation process in the latter stages and water loss due to evaporation during the reaction, which reduced the density at high temperature.

Figure 6 also represented the influence of exposure to the elevated temperature on the compressive strength of metakaolin based alkali activated materials. The results obtained ranged from 10.338 MPa to 20.975 MPa; 20.975 MPa was the highest compressive strength at 60 °C curing temperature, while 10.338 MPa was the lowest compressive strength after being exposed at 900 °C. In general, the compressive strength was slightly increased from ~25 °C to 60 °C, and then fluctuated at 100 °C up to 900 °C. These elevated temperatures resulted in physical and chemical transformations in the alkali activated materials. As the curing temperature was set at ~25 °C, after curing for 24 h, it was not sufficient to remove the unconjugated water, and the alkali activated material slurries were still gelatinous and moist. Therefore, the compressive strength was lower because of the plentifully existing sol phase and water in the system. This statement was also discussed by Azevedo et al. [72]. As the temperature rose up to 900 °C, the microcracks became wider and spread throughout the whole surface of the specimen, resulting in a decrease in strength. Thermal cracking was caused by a thermal mismatch in the alkaline activator metakaolin, which caused microcracks to develop. From a compressive strength point of view, curing at 60 °C for 24 h revealed the optimum strength for metakaolin based alkali activated materials.

#### 3.3.3. Microstructure Analysis

**Figure 7:**
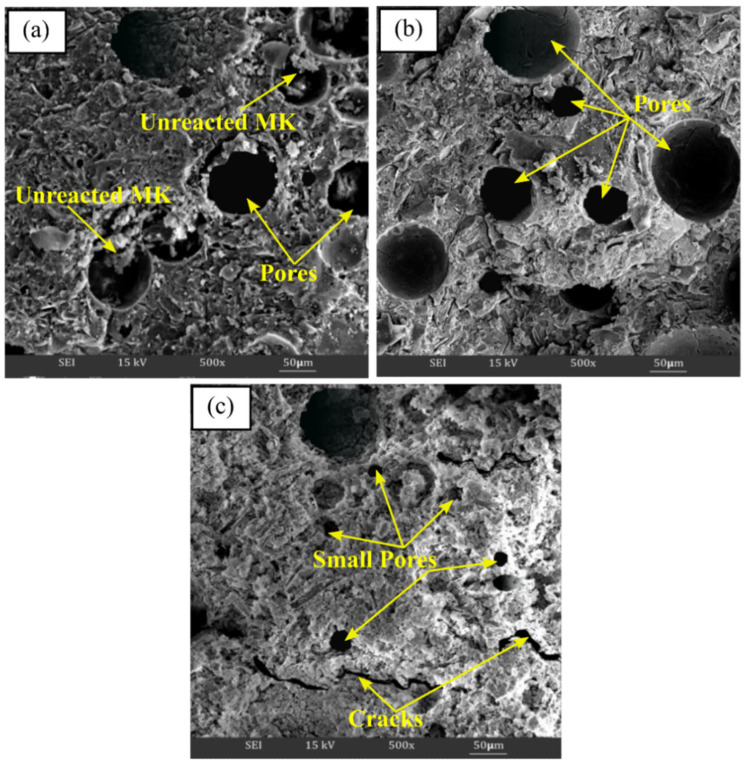
SEM micrograph of metakaolin based alkali activated materials at (**a**) room temperature, (**b**) 60 °C, and (**c**) 900 ℃ exposure temperature.

SEM analysis clarified the microstructural or surface structure changes that occurred when the samples were exposed to different temperatures, as depicted in Figure 7. As can be seen, alkali activation products consist of a compacted alkali activated materials matrix, a number of unreacted metakaolin particles, pores in various scales, and the formation of cracks at room temperature curing conditions, as can be seen in Figure 7a. However, the microstructure findings revealed that when the exposing temperature was raised, the quantity of unreacted metakaolin particles was reduced. Up to 60 °C, the matrix in Figure 7b seems heterogeneous, and more porous, and becomes denser as the aluminosilicate gel has completely formed. According to Kong et al. [73] and Tamjidi et al. [74], adsorbents with larger and wider porous surfaces capture more heavy metal ions, enhancing the potential of the material for sorption.

In addition, the microstructure of metakaolin that has been alkali activated at higher temperature (900 °C) is shown in Figure 7c. Typically, higher exposing temperatures resulted in the appearance of the alkali activated metakaolin structure with a sponge-like structure and smooth conglomerated surface. The thermal treatment temperature subsequently caused visible cracking in the metakaolin-based alkali activated materials, as well as irregularly shaped pores on the surface due to pore size shrinkage (Figure 7c). The appearance of cracks in the structure was due to the loss of water with rising temperature exposure. Therefore, the strength of alkali activated metakaolin decreased, as shown in Figure 6.

#### 3.3.4. Phase Analysis

**Figure 8:**
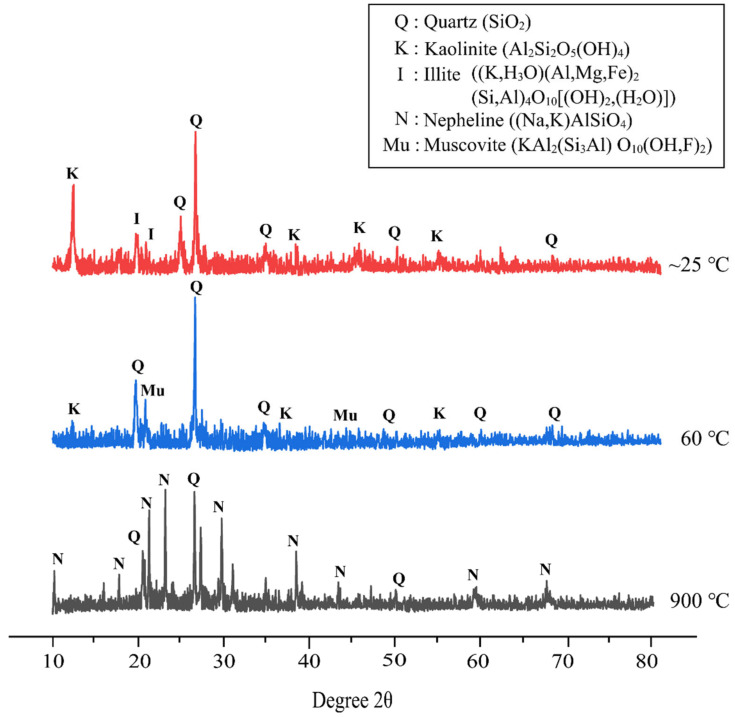
XRD patterns of metakaolin based alkali activated materials at different temperature exposures.

Figure 8 demonstrates the XRD pattern of metakaolin based alkali activated materials at different temperature exposures. The XRD pattern of alkali activated metakaolin at elevated temperatures with the best design shows quartz (SiO_2_) as the major mineral in all samples. This reflection of quartz serves as the primary component of the flake-like structure of metakaolin as a precursor detected by SEM, as discussed before. Overall, the amorphous phase dominates the XRD pattern of metakaolin-based alkali activated materials cured at room temperature and 60 °C. The amorphous halo and position were roughly the same, but the halo peak’s height was reduced. This might indicate a nanostructural change, such as gel disintegration or dissolution.

Samples cured at 60 °C showed a content of quartz (SiO_2_) (ICDD reference: 01-083-0539) as the major mineral at 2θ values of 26.593 °, and some minor minerals of muscovite (KAl_2_(Si_3_Al) O_10_(OH,F)_2_) (ICDD reference: 01-075-0948) at 2θ values of 22.990° and 45.214°, and kaolinite (Al_2_Si_2_O_5_(OH)_4_) (ICDD reference: 01-075-1593) at 12.402°, 38.558°, and 55.517°. As per Salam et al. [75], Zhou et al. [76], and Xu et al. [77], muscovite is one of the micaceous minerals that are highly degraded in nature and are good adsorbents of metals. This was attributed to muscovite having perfect cleavage that allows the creation of large areas of smooth surface in the atomic scale.

Further, the major peak of quartz crystal (ICDD reference: 01-085-1780) for the metakaolin-based alkali activated materials exposed at 900 °C shifts to a lower angle, whereas the other crystal phases gradually fade or even disappear. Due to thermal shrinkage, the amorphous phase of metakaolin-based alkali activated materials becomes increasingly resistant to crystallization as the exposure temperature rises [78]. Thus, increasing the exposed temperature caused densification to decrease, as seen in Figure 6. According to Liu et al. [79], quartz crystals are difficult to decompose, and only translate into polymorphism due to their high lattice energy. The alkali activated metakaolin was then annealed and transformed into nepheline ((Na,K)AlSiO_4_) (ICDD reference: 01-076-2469), which was subsequently used in other applications. New crystallization peaks appeared at 18.588°, 20.509°, 23.118°, 29.666°, 38.343°, 43.178°, 59.100°, and 67.540°. This research supports the findings of Zawrah et al. [80] and Lahoti et al. [81], who found that raising the sintering temperature increase the quantity of nepheline that is formed.

#### 3.3.5. Nitrogen Adsorption–Desorption Isotherms Analysis

**Figure 9:**
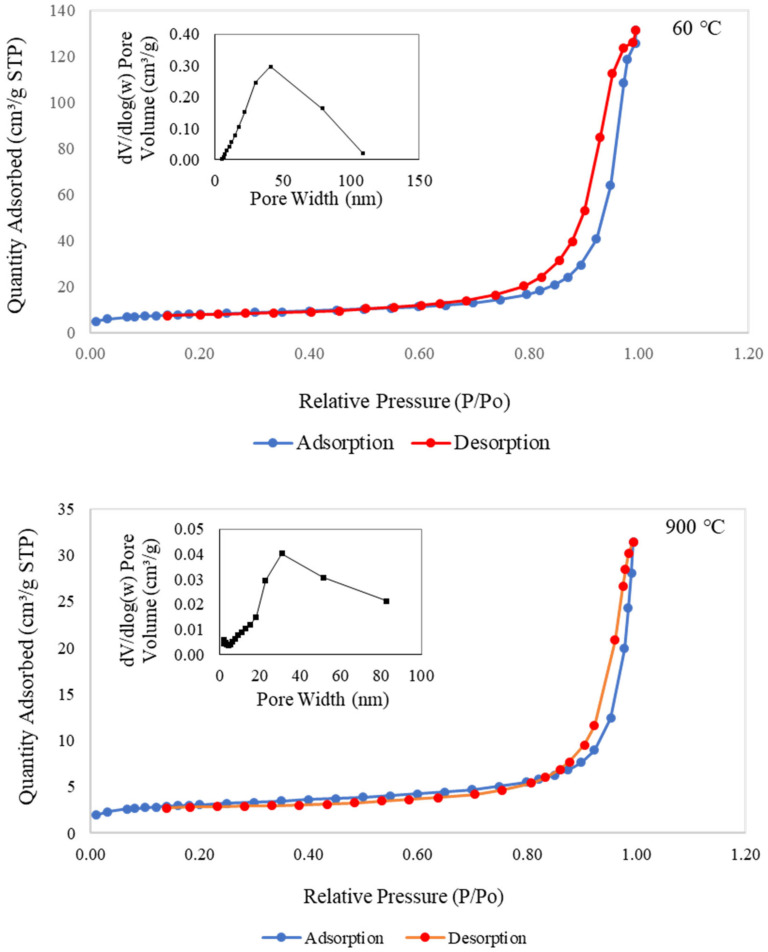
Nitrogen adsorption–desorption isotherms and pore size distribution curve of the metakaolin based alkali activated materials exposed at 60 °C and 900 °C.

Figure 9 presents the nitrogen adsorption–desorption isotherms and pore size distribution for metakaolin-based alkali activated materials cured at 60 °C and subjected to a higher temperature of 900 °C. Both samples displayed adsorption–desorption isotherms of type IV with limited multilayer development and typed H2 hysteresis loops for validating the porosity of the regular channel structure. These considerations highlighted the increasing boundary curve in the isotherm plot, according to the corresponding area that was connected with the porous silica associated with opening pores and with homogenous ordered frameworks [16,82,83,84]. The metakaolin-based alkali activated materials had higher specific area, S_BET_, and total pore volume, V_T_, values than the raw material, as shown in Table 2. According to the BET technique, the S_BET_ values for raw materials and samples of metakaolin that were cured at 60 °C and exposed at 900 °C were 4.575, 24.608, and 9.669 m^2^/g, respectively, with the V_T_ values of 0.0124, 0.1716, and 0.0283 cm^3^/g, respectively.

Additionally, samples exposed at 900 °C had maximum pore sizes between 20 and 40 nm, whereas samples cured at 60 °C had maximum pore sizes between 40 and 50 nm. It is obvious from SEM morphological analysis in Figure 7c that the pores began to shrink, resulting in smaller pores. This finding indicates that 60 °C curing of metakaolin-based alkali activated materials results in more uniform pore size distribution with greater micro and mesoporosity. The findings also showed that the metakaolin-based AAMs had greater active areas, pore volumes, and pore sizes when exposed at lower temperatures. This great pore structure of micro/mesopore design is expected to provide higher copper adsorption capacity and copper removal efficiency to adsorbents due to larger surface area and improved interaction between the diffusing molecules and the surface.

#### 3.3.6. Elemental Distribution from Micro-XRF Analysis

**Figure 10:**
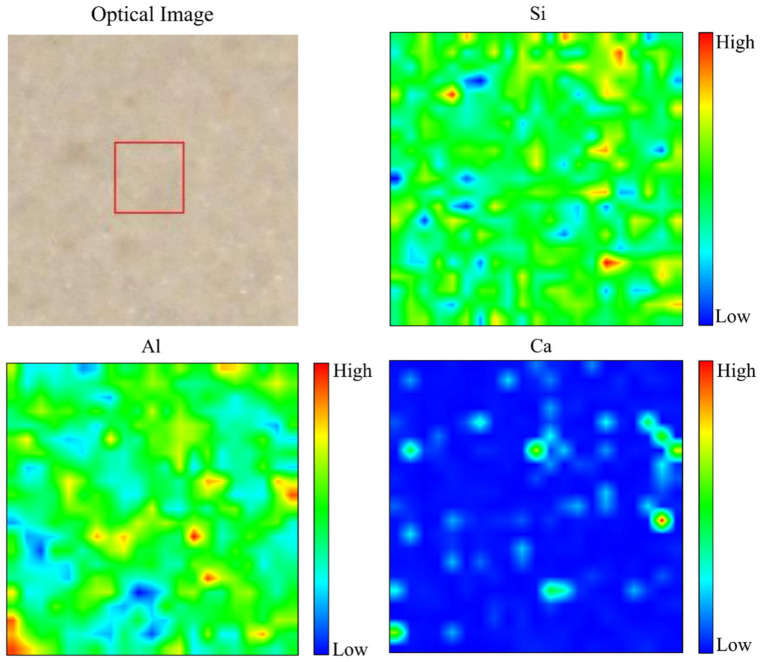
Optical image and micro-XRF elemental distribution maps of Si, Al, and Ca of metakaolin-based alkali activated materials at 60 °C.

The distribution of Si, Al, and Ca, with optical images, in metakaolin-based alkali activated materials that were cured at 60 °C for 24 h is presented in the micro-XRF elemental maps of Figure 10. Due to the maximum water absorption with regular pore distribution from the microstructure image, 0.5 Na_2_SiO_3_/NaOH cured at 60 °C to metakaolin based alkali activated materials as an adsorbent was chosen for micro-XRF study. High concentration is shown by a reddish colour tone on the colour bar, whereas a blueish colour tone represents low concentration. A high concentration of Si shows homogenous distribution due to the phases (quartz, kaolinite and muscovite) present, which consists of the Si element. A high concentration of Al indicates the phases of kaolinite and muscovite in these samples. In line with chemical composition (Table 1) and phase analysis results presented in Figure 8, it was indicated that the distribution of Ca was limited on metakaolin-based alkali activated materials by examining at the elemental distribution from micro-XRF analysis, which displayed mostly blueish colour tone and a little amount of greenish colour.

### 3.4. Adsorption of Copper Ions Analysis

**Figure 11:**
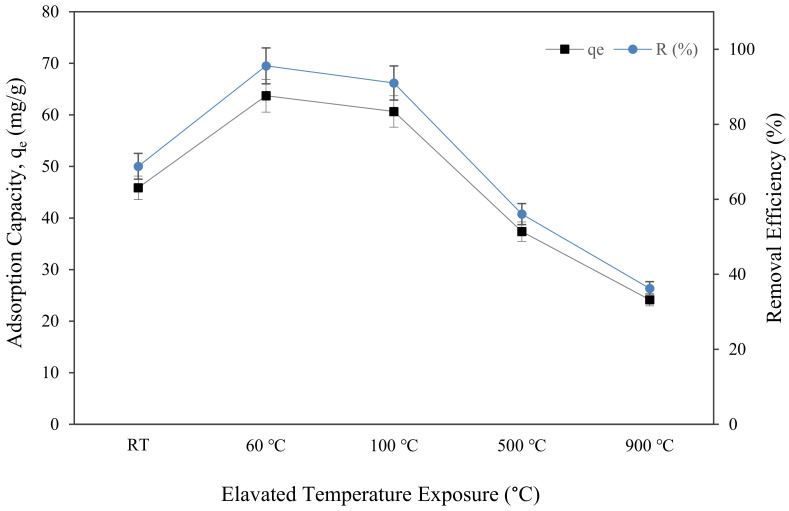
Adsorption capacity and removal efficiency of copper ions by metakaolin based alkali activated materials adsorbent at different elevated temperatures at adsorption conditions of pH = 5, a dosage of 0.15 g, the temperature of 25 °C, and contact time of 60 min.

Figure 11 depicts the uptake of Cu (II) metal ions, q_e_, and copper removal efficiency, R (%), attained by metakaolin-based alkali activated subjected to elevated temperature. The maximum copper ion uptake was found in metakaolin based alkali activated materials, which were only cured at 60 °C, and had a 63.73 mg/g adsorption capacity and 95.59% copper ion removal efficiency at optimal conditions of pH = 5, the dosage of 0.15 g, the temperature of 25 °C, and contact time of 60 min. This finding was comparable to that reported by Tan et al., who found that the Q_e_ of Cu (II) by porous metakaolin geopolymer adsorbents was around 35.5 mg/g under the same adsorption condition, yet required 50 h of contact time to achieve the greatest copper ion absorption [35]. It is evident from the graph that, following an hour of adsorption, the sorption of copper ions and copper removal efficiency initially increased from the sample at room temperature to curing at 60 °C, going from 45.864 mg/g to 63.726 mg/g of q_e_ and from 68.7% to 95.59% of copper removal efficiency. However, when exposed to the highest temperature, 900 °C, the q_e_ and R (%) dropped to the lowest points, at 24.164 mg/g and 36.25%, respectively. The highest copper removal efficiency at samples cured for 60 °C means that the metakaolin-based alkali activated material surface structure modification at this temperature had more Cu^2+^ ion sorption sites and physical characteristics than the samples subjected to higher temperatures. This investigation agreed with the findings of previous researchers who found that the volume of large pores and medium pores reduced in materials subjected to high temperature due to shrinking of the lattice structures and complicated thermal expansion behaviour that changes with time and pore system moisture [85,86,87]. The adsorbents physical characteristics, such as pore size, pore volume, and specific surface area, as illustrated in Figure 7b and Figure 9, are additional crucial elements that significantly affect the absorption process.

## 4. Conclusions

The utilization of metakaolin as the main source for alkali activated materials as an adsorbent, with a variety of mixing design preparations, significantly influences the properties of the fresh and hardened states of alkali activated materials and their porosity structure. This study’s conclusion can be drawn based on the study objectives. The ratio 0.5 Na_2_SiO_3_/NaOH is the optimum ratio, which resulted in the highest water absorption, lowest density, and acceptable compressive strength to be an adsorbent. A higher sodium silicate to sodium hydroxide ratio is not recommended due to the higher viscosity of a higher Na_2_SiO_3_/NaOH ratio, which turns to more extensive bubble deformation and faster bubble break up into smaller bubbles that produce lower water absorption. In addition, curing and exposure to high-temperature conditions also play a crucial role in achieving the optimum properties of water absorption percentage, and also for their microstructure performances that need to be considered as an effective adsorbent for alkali activated metakaolin. It was discovered that the metakaolin based alkali activated materials cured at 60 °C with 63.73 mg/g adsorption capacity and 95.59% copper removal efficiency had the greatest mechanical and physical properties, with good microstructural performances required for an effective adsorbent. High heat temperature exposure was not effective, which attributed to the reduction of water absorption percentage due to high heat shrinkage, which caused the hardened structure to become more compact, lowering its permeable porosity. The pore size distribution, the pore connectivity, the shape, and also the volume of the pore space are very important factors that govern the water permeability of alkali activated metakaolin paste. In addition, amorphous foaming porous alkali activated metakaolin leads to the formation of a porous structure, encouraging Cu^2+^ adsorption. Thus, the metakaolin-based alkali activated materials adsorbent was created using a straightforward procedure and was synthesized at a temperature of 60 °C, which is lower than the sintering temperature for typical conventional zeolite, adsorbents, or membranes used for heavy metals removal, which require heat treatment up to 1600 °C depending on the material type. These considerations have increased the requirement for environmentally sustainable adsorbents at free or lower sintering temperature that remove copper ions efficiently.

## Figures and Tables

**Table 1:** Chemical composition of metakaolin.

Number	Component	Wt%
1	Al_2_O_3_	34.7
2	SiO_2_	56.7
3	P_2_O_5_	1.68
4	K_2_O	0.607
5	CaO	0.700
6	TiO_2_	3.13
7	Fe_2_O_3_	2.09
8	CuO	0.0383
9	Ga_2_O_3_	0.0398
10	SrO	0.0530
11	ZrO_2_	0.199

**Table 2:** Specific surfaces area and total pore volume of metakaolin based alkali activated materials.

Sample	Specific Surface Area, S_BET_ (m^2^/g)	Total Pore Volume, V_T_ (cm³/g)
MK	4.475	0.012366
60 °C	24.608	0.171588
900 °C	9.669	0.028246

## Data Availability

Not applicable.
